# Membranous nephropathy in a patient with hereditary angioedema: a case report

**DOI:** 10.1186/1752-1947-2-328

**Published:** 2008-10-13

**Authors:** Sandawana W Majoni, Steven R Smith

**Affiliations:** 1 Russells Hall Hospital Renal Unit, Dudley Group of Hospitals NHS Trust, Dudley, West Midlands, UK

## Abstract

**Introduction:**

Hereditary angioedema is the commonest inherited disorder of the complement system and has been associated with several immune glomerular diseases. A case of nephrotic syndrome and renal impairment due to idiopathic membranous glomerulonephritis in a patient with hereditary angioedema has not been described before.

**Case presentation:**

We present the first reported case of the association of membranous nephropathy and hereditary angioedema in a 43-year-old male Caucasian patient who presented with acute intestinal angioedema, hypertension, acute pancreatitis, renal impairment and generalised body swelling due to severe nephrotic syndrome. We present the challenges involved in the clinical management of the patient.

**Conclusion:**

This patient's presentation with severe nephrotic syndrome, renal impairment and hypertension required aggressive treatment of the membranous nephropathy given the high risk for progression to end stage renal failure. The contraindication to angiotensin converting enzyme inhibitors and angiotensin II receptor blockers in this patient, the lack of published evidence on the use of alkylating agents and other immunosuppressive agents in patients with hereditary angioedema and the lack of published data on the management of similar cases presented a clinical challenge in this patient's management.

## Introduction

Hereditary C1 esterase inhibitor deficiency (hereditary angioedema; HAE) is a rare (incidence 1 in 10,000 to 1 in 150,000) autosomal dominant inherited disease of the complement system characterised by the absence or dysfunction of the protein C1 esterase inhibitor (C1 INH), which regulates the complement, fibrinolytic, coagulation and kinin cascades [1]. It is the commonest inherited disorder of the complement system which is characteristically associated with non-pruritic angioedema, most commonly affecting the respiratory system, the skin and the gastrointestinal tract [1]. It has been associated with other immunoregulatory disorders (Table 1).

**Table 1:** Types of HAE and some associated immunoregulatory disorders

Type	Characteristics	Comments	Some immunological conditions associated with all types of HAE
1	Low or absent C1 esterase inhibitor activity	Autosomal dominant. Constitutes 80–85% of cases	Systemic lupus erythematosus, mesangiocapillary glomerulonephritis, autoimmune thyroiditis, rheumatoid arthritis, urticaria, other glomerulonephritides, Sjögren's syndrome, coagulopathies
2	Normal or raised activity of a dysfunctional C1 esterase inhibitor	Autosomal dominant. Constitutes 15–20% of the cases	
3	Normal C1 esterase inhibitor level and function	X linked dominant newly described in women	

The association of HAE with membranous glomerulonephritis has not been reported before, as we far as we know. The management of membranous glomerulonephritis in a patient with HAE would be challenging as angiotensin converting enzyme inhibitors (ACEIs) and angiotensin 2 receptor blockers (ARBs) which effectively reduce proteinuria and slow the progression of the renal disease [2] cause angioedema which precludes their use in patients with HAE [3]. Although alkylating agents such as chlorambucil and cyclophosphamide and the immunosuppressant cyclosporin are effective in the treatment of membranous nephropathy [2], their safety in a patient with HAE is unknown. The effect of renal failure on HAE and vice versa is also unknown. We report a case of nephrotic syndrome and renal failure due to membranous glomerulonephritis in a patient with HAE.

## Case presentation

A 43-year-old Caucasian man was first diagnosed with hereditary angioedema in 1982 after admission to the intensive care unit with acute airway obstruction. Investigations were consistent with type 1 HAE, showing low C1 esterase inhibitor activity of 0.06 g/litre (normal range [NR] 0.2 to 0.65 g/litre), low complement C4 and normal complement C3. He was discharged on 17alpha-ethinyl testosterone (Danazol). He had had recurrent tonsillitis and abdominal pain from the age of 4 years leading to tonsillectomy and appendicectomy. He did not know his biological family. He had three further admissions with abdominal pain in the 1990s followed by full recovery after treatment with subcutaneous adrenaline and fresh frozen plasma.

He presented to our hospital in 2001 with acute abdominal pain and generalised body swelling. Clinical examination showed pallor, generalised oedema and abdominal tenderness. His blood pressure was 146/90 mmHg. He had bilateral pleural effusions which were confirmed by chest radiography. The rest of the examination was unremarkable. Urine dipstick was positive for protein, nitrates, leucocytes and a trace of blood. Urine culture was negative. Serum creatinine and serum albumin were 148 μmol/litre and 13 g/litre, respectively. 24 hour urine protein excretion was 6.3 g. Serum amylase was elevated (340 IU/litre [NR 35 to 110 U/l]). Serum lipids were also raised. Haemoglobin and erythrocyte sedimentation rate (ESR) were 10 g/dl and 80 mm in the first hour (NR < 20 mm), respectively. The following autoimmune serological tests were negative: antineutrophil cytoplasmic antibodies, antinuclear antibodies, extractable nuclear antigen antibodies, other lupus serology, antiglomerular basement membrane antibodies and rheumatoid factor. Hepatitis screen (hepatitis B and C), liver function tests, serum protein electrophoresis, C-reactive protein (CRP) and all his other blood results were normal.

**Figure 1:**
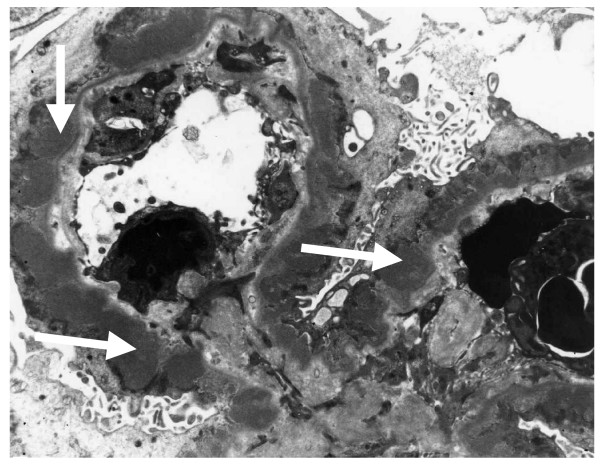
Stage 3 membranous glomerulonephritis with medium-sized subepithelial dense deposits and basement membrane reaction surrounding most of the deposits (arrows) (transmission electron microscopy, original magnification ×11,000).

Ultrasound scan showed normal sized kidneys and ascites, findings confirmed by computerised tomography (CT) scan. The CT also confirmed acute pancreatitis and bowel oedema. A renal biopsy performed 4 days after diuretic treatment to reduce the oedema showed stage 3 membranous glomerulonephritis (Figure 1).

In conclusion, the patient thus had mild pancreatitis, acute intestinal angioedema and nephrotic syndrome and moderate renal impairment due to membranous glomerulonephritis.

He was treated with infusion of C1 INH concentrate, reducing course of prednisolone, starting at 60 mg daily slowly tapering to 10 mg daily over 2 weeks, bisoprolol 5 mg once daily as well as furosemide 80 mg daily reducing to 40 mg daily. His abdominal pain resolved within 24 hours and serum amylase normalised after 3 days. He was discharged 7 days later with serum creatinine of 168 μmol/litre and on losartan potassium which he tolerated. Proteinuria improved to 2 g per day within 2 weeks of discharge. He had no further attacks of angioedema for 4 weeks.

He was readmitted 4 weeks later with worsening nephrotic syndrome and abdominal pain and a rise of serum creatinine to 415 μmol/litre. Serum albumin was 10 g/litre while proteinuria was 10 g/day. The losartan was stopped. He was given an infusion of C1 INH concentrate and increased doses of prednisolone and furosemide. Cyclosporine was added. He was discharged 10 days later with serum creatinine and proteinuria of 293 μmol/litre and 2 g/day, respectively.

He remained stable on daily prednisolone 5 mg, furosemide 40 mg, bisoprolol 10 mg and cyclosporine 100 mg twice daily. At follow-up in the low clearance clinic 4 weeks later, proteinuria had improved to less than 1 g per day. Serum creatinine was 250 μmol/litre. He remained off the danazol because of the renal impairment.

## Discussion

The association of HAE with immunoregulatory disorders is well documented (Table 1). The commonest reported glomerular diseases associated with HAE are lupus nephritis and mesangiocapillary glomerulonephritis. Brickman *et al. *[4] evaluated 157 patients manifesting features of autoimmunity. Nineteen patients had clinical immunoregulatory disorders of which five had glomerulonephritis, the majority of which were mesangiocapillary glomerulonephritis. Pan *et al. *[5] reported long-term follow-up of four cases of non-SLE glomerulonephritis, none of whom developed renal failure after 8 to 25 years of follow-up. Three members of the same family with HAE associated with IgA nephropathy have also been reported [6]. To our knowledge, the association of HAE and membranous nephropathy has not been previously reported.

Our patient had had HAE for about 20 years before he presented with nephrotic syndrome. The HAE classically manifested in childhood and led to tonsillectomy and appendicectomy before the diagnosis, which is typical since with no suggestive family history, the condition can be misdiagnosed leading to patients having unnecessary surgery [1]. His presentation with recurrent abdominal pain and respiratory problems, due to intestinal and upper airway angioedema, respectively, is typical. Acute pancreatitis is a recognised complication of the condition [1].

This case illustrates some of the challenges which may be involved in managing renal impairment and nephrotic syndrome due to membranous nephropathy in a patient with HAE. There are not much data on the effect of renal failure on HAE and, since angioedema causes fluid retention, this may complicate the management of fluid overload states in these patients. It may be difficult to distinguish between fluid overload and attacks of angioedema in patients with HAE and renal failure or nephrotic syndrome [7]. Ohsawa *et al. *described a case with end stage renal disease due to membranoproliferative glomerulonephritis (MPGN) who had difficult worsening fluid retention. This case illustrates the difficulties in controlling fluid overload as the attacks of angioedema worsened the fluid retention due to the end stage renal disease and nephrotic syndrome as in our patient [8].

Our patient had no evidence of SLE or any other immunological condition. His presentation with membranous nephropathy causing severe nephrotic syndrome, hypertension and renal impairment indicated a high risk for progression to end stage renal disease [2]. He would, therefore, require aggressive treatment. There is clear evidence that ACEIs and/or ARBs effectively reduce proteinuria and delay the progression to end stage renal disease in membranous nephropathy [2]. However, by interfering with the contact (kallikrein-kinin) system, ACEIs cause angioedema and are thus contraindicated in patients with any history of angioedema [3].

When ARBs were first used in clinical practice, they were thought to provide an alternative for use in people with ACEI induced cough and angioedema since they do not directly interfere with the contact (kallikrein-kinin) system. At the time this patient presented, there had been sporadic case reports of suspected ARB induced angioedema leading to advice for their use with caution in patients with a previous history of angioedema. Our assumption at that time was that losartan would be worth trying in this patient. Though the patient may have responded to the losartan given the initial improvement in proteinuria and renal function, he developed acute intestinal angioedema which improved on stopping the losartan and giving him C1 INH concentrate infusion. There have since been many more reported cases of angioedema associated with ARBs [9], hence their contraindication in people with HAE.

## Conclusion

Given the high risk for progression to end stage renal failure in this patient, the treatment of the membranous nephropathy would require aggressive control of his blood pressure using antihypertensive drugs other than the contraindicated ACEIs and ARBs. He would also require alkylating agents such as chlorambucil and cyclophosphamide or the immunosuppressant cyclosporine [2] for the management of the membranous nephropathy.

The emergency treatment of HAE includes purified C1 INH concentrate infusion or fresh frozen plasma (contains C1 esterase inhibitor) and/or subcutaneous adrenaline. Corticosteroids and antihistamines are ineffective. For long-term prophylaxis, attenuated androgens such as 17alpha-ethinyl testosterone (Danazol) and stanozolol potent androgens such as oxandrolone and antifibrinolytic agents such as tranexamic acid and epsilon aminocaproic acid are effective [10] but their safety in patients with advanced renal disease is unclear.

Our patient required an increasing dosage of 17alpha-ethinyl testosterone (Danazol) as the frequency of the attacks of angioedema increased. However, with the development of advanced renal disease; he required more careful monitoring of the prophylactic treatment. He tolerated the cyclosporine [11], which we believe, has led to the improvement in the proteinuria and renal function. The prognosis of HAE is generally good with treatment [1]. However, the membranous nephropathy causing renal impairment has worsened the overall prognosis of this patient who will most likely require renal replacement therapy in the future. The effect of dialysis and or renal transplantation on HAE will need to be carefully assessed. Currently, there are few data in the literature to inform the best management of this patient.

## Abbreviations

ACEI: angiotensin converting enzyme inhibitor; ARBs: angiotensin receptor blockers; CT: computed tomography; C1 INH: CC1 esterase inhibitor; ESR: erythrocyte sedimentation rate; HAE: hereditary angioedema; NR: normal reference range; SLE: systemic lupus erythematosus

## Competing interests

The authors declare that they have no competing interests.

## Authors' contributions

SWM collected the data and prepared the first draft of the manuscript. SRS revised the manuscript and contributed equally to the final draft. Both SRS and SWM examined and reviewed the renal biopsy histology with the pathology department. All authors read and approved the final draft.

## Consent

Written informed consent was obtained from the patient for publication of this case report and any accompanying images. A copy of the written consent is available for review by the Editor-in-Chief of this journal.

## References

[B1] Nzeako UC Frigas E Tremaine WJ Hereditary angioedema: a broad review for clinicians Arch Intern Med 2001 161 20 2417 2429 10.1001/archinte.161.20.2417 11700154

[B2] Cattran D Management of membranous nephropathy: when and what for treatment J Am Soc Nephrol 2005 16 5 1188 1194 10.1681/ASN.2005010028 15800117

[B3] Sabroe RA Kobza Black A Angiotensin-converting enzyme (ACE) inhibitors and angio-oedema Br J Dermatol 1997 136 2 153 158 10.1111/j.1365-2133.1997.tb14887.x 9068723

[B4] Brickman CM Tsokos GC Balow JE Lawley TJ Santaella M Hammer CH Frank MM Immunoregulatory disorders associated with hereditary angioedema: I. Clinical manifestations of autoimmune disease J Allergy Clin Immunol 1986 77 5 749 757 10.1016/0091-6749(86)90424-0 3084606

[B5] Pan CG Strife CF Ward MK Spitzer RE McAdams AJ Long-term follow-up of non-systemic lupus erythematosus glomerulonephritis in patients with hereditary angioedema: Report of four cases Am J Kidney Dis 1992 19 6 526 531 1595700 10.1016/s0272-6386(12)80830-4

[B6] Srinivasan J Beck P IgA nephropathy in hereditary angioedema Postgrad Med J 1993 69 808 95 99 8506211 10.1136/pgmj.69.808.95 PMC2399629

[B7] Nomura H Tsugawa Y Koni I Tofuku Y Mabuchi H Takeda R Sato T Hereditary angioedema complicated with chronic renal failure: Report of sibling cases Intern Med 1992 31 1 94 97 10.2169/internalmedicine.31.94 1568052

[B8] Ohsawa I Satomura A Fuke Y Hidaka M Endo M Fujita T Ohi H Worsening fluid retention in a patient with hereditary angioedema and end stage renal disease Intern Med 2004 43 8 708 712 10.2169/internalmedicine.43.708 15468971

[B9] Adachi YU Iwakiri S Katoh T Angioedema, angiotensin converting enzyme inhibitors, and angiotensin receptor blocking drugs Can J Anaesth 2007 54 2 155 157 17272257 10.1007/BF03022016

[B10] Fay A Abinun M Current management of hereditary angio-oedema (C'1 esterase inhibitor deficiency) J Clin Pathol 2002 55 4 266 270 11919209 10.1136/jcp.55.4.266 PMC1769636

[B11] Cattran DC Alexopoulos E Heering P Hoyer PF Johnston A Meyrier A Ponticelli C Saito T Choukroun G Nachman P Praga M Yoshikawa N Cyclosporin in idiopathic glomerular disease associated with the nephrotic syndrome: Workshop recommendations Kidney Int 2007 72 12 1429 1447 10.1038/sj.ki.5002553 17898700

